# An online forum for psychiatric patients: Sexual life and self-harm

**DOI:** 10.1192/j.eurpsy.2025.2368

**Published:** 2025-08-26

**Authors:** E. B. Lyubov, N. Semenova

**Affiliations:** 1 Moscow Research Institute of Psychiatry – a branch of the V. Serbsky National Medical Research Centre for Psychiatry and Narcology of the Ministry of Health of the Russian Federation, Moscow, Russian Federation

## Abstract

**Introduction:**

The significance of intimate life in a person’s personal and social experience cannot be overstated. This aspect is intricately woven into the clinical and internal picture of intentional (suicidal or non-suicidal) self-harm (SX), making it a crucial area of study.

**Objectives:**

Study of position on the issue of sexual problems and SX among participants of an open forum.

**Methods:**

We conducted interviews with forty participants (90% women) with a mean age of 27 (range 17-43) years, using an original semi-structured questionnaire. This approach allowed us to delve into the issue of sexual problems and SX among participants of an open forum.

**Results:**

Тen thousand people were interested in the survey during two months of posting on the site, but < 0.5% participated. All questions were answered by 95%. The sample comprised young women, like most of our online polls. For almost 70% of respondents, issues of sexual life are burning and pressing questions, but only ¼ discuss «this» with a psychiatrist and only in terms of drug side effects. During routine visits, the doctors were not interested in the sexual life of about 80% of patients; ¾ of the women were not interviewed about the menstrual cycle or (all men) about erections or galactorrhea. Only ¼ of doctors are interested in the intent of SX. >70% indicate that forced abstinence «worsens» their mental state (makes them irritable), >¾ of singles and ½ family people masturbated 1-3 times a week («to feel alive»). >½ of married women are afraid of pregnancy, but refuse birth control as «harmful». >80% of family problems were associated with apathy and irritability and are considered the main obstacle to regular sex. > ½ report «habitual» weakness, lack of interests (including sexual), and suicidal thoughts for more than three months. <½ respondents link treatment to impotence and frigidity. ½ had SX experience (indistinguishable from suicide attempts) due to the loss of a loved one during their life. Patients considered SX to be a «natural» reaction to shame and mental pain and allowed repetition in a similar situation. Doctors do not regularly assess a patient’s risk of suicide.

**Conclusions:**

The findings of this study underscore the need to bring sex and SX topics into the focus of routine psychiatric practice. The passive role of psychiatrists in clarifying patients’ problems that influence treatment adherence, well-being, and undertreatment of depression is a significant issue that needs to be addressed. The survey highlights two subtopics of greatest interest to patients: indestructible sex drive and micro social problems as ‘reasons’ for depression and SX. The ‘gray zone’ of therapeutic contact and satisfaction with care is an area that warrants further exploration.

**Disclosure of Interest:**

None Declared

